# Novel ultra-low temperature co-fired microwave dielectric ceramic at 400 degrees and its chemical compatibility with base metal

**DOI:** 10.1038/srep05980

**Published:** 2014-08-07

**Authors:** Zhou Di, Pang Li-Xia, Qi Ze-Ming, Jin Biao-Bing, Yao Xi

**Affiliations:** 1 Electronic Materials Research Laboratory, Key Laboratory of the Ministry of Education & International Center for Dielectric Research, Xi'an Jiaotong University, Xi'an 710049, Shaanxi, China; 2 Micro-optoelectronic Systems Laboratories, Xi'an Technological University, Xi'an 710032, Shaanxi, China; 3 National Synchrotron Radiation Laboratory, University of Science and Technology of China, Anhui, 230029, Hefei, China; 4 Research Institute of Superconductor Electronics (RISE), School of Electronic Science and Engineering, Nanjing University, Nanjing, Jiangsu, 210093, China

## Abstract

A novel NaAgMoO_4_ material with spinel-like structure was synthesized by using the solid state reaction method and the ceramic sample was well densified at an extreme low sintering temperature about 400°C. Rietveld refinement of the crystal structure was performed using FULLPROF program and the cell parameters are a = b = c = 9.22039 Å with a space group F D −3 M (227). High performance microwave dielectric properties, with a permittivity ~7.9, a Qf value ~33,000 GHz and a temperature coefficient of resonant frequency ~−120 ppm/°C, were obtained. From X-ray diffraction (XRD) and Energy Dispersive Spectrometer (EDS) analysis of the co-fired sample, it was found that the NaAgMoO_4_ ceramic is chemically compatible with both silver and aluminum at the sintering temperature and this makes it a promising candidate for the ultra-low temperature co-fired ceramics technology. Analysis of infrared and THz spectra indicated that dielectric polarizability at microwave region of the NaAgMoO_4_ ceramic was equally contributed by ionic displasive and electronic polarizations. Its small microwave dielectric permittivity can also be explained well by the Shannon's additive rule.

Microwave dielectric ceramics have been widely used as the dielectric resonator, filter, substrate, capacitor etc. in the past half century^1^,^2^. Most microwave dielectric ceramics are inorganic, poly-crystal oxides, such as the popular Al_2_O_3_, BaO-TiO_2_ etc. Usually, the ceramic materials are strong in compression, weak in shearing and tension. Recent years due to the fast development of the electronic devices, the organic has become hot while the ceramic has become “colder”. However, some central passive devices can only be made using ceramic materials. Low temperature co-fired ceramic (LTCC) technology has played an important role in the fabrication of modern electronic devices^3^,^4^. Advantage of this technology is that the green tape, consisting of inorganic powders and organic materials, is soft and easy to be processed layer by layer separately in the meantime, and finally passive components can be integrated within a monolithic bulk module with IC chips mounted on the surface. Appropriate permittivity (ε_r_), high quality factor (Qf), and lower sintering temperature (S. T.) than the melting point of inner metal electrode are required for the dielectric ceramics to meet LTCC technology.

The most popular method to lower sintering temperature of dielectric ceramic with high microwave dielectric performance is the addition of glass or low melting point oxides (B_2_O_3_ based, SiO_2_ based etc.)^3^. The addition usually caused the deterioration of Qf value. In the past decade, a so-called ultra-low temperature co-fired ceramic (ULTCC), which is focused on the microwave dielectric ceramic with intrinsic low sintering temperature, has started a new stage for the LTCC technology. The two classic examples are BaTe_4_O_9_ (ε_r_ = 17.5, Qf = 54,700 GHz and S. T. = 550°C) and Bi_2_Mo_2_O_9_ (ε_r_ = 38, Qf = 12,500 GHz and S. T. = 620°C) ceramics, which was reported in 2005 and 2008, respectively^5^,^6^. Both of them are chemically compatible with aluminum, which means that Al can be used as the inner electrode. The search of novel ULTCC in the low eutectic points Mo-rich and Te-rich compounds has attracted more and more attention^7^,^8^. In the present work, a novel spinel-like compound NaAgMoO_4_ with an extreme low sintering temperature about 400°C was reported. The preparation, sintering behavior, microwave dielectric properties and chemical compatibility with both silver and aluminum were studied in detail.

Figure 1 (a) shows XRD patterns of the NaAgMoO_4_ samples calcined at 350°C/4 h, sintered at 400°C/4 h, co-fired with 30 wt. % silver at 400°C/4 h and 30 wt. % aluminum at 450°C/4 h. It is seen that single phase with spinel-like structure was formed after calcinations at 350°C. However, a weak trace of silver is observed at 38.1° and it may be caused by decomposition of the Ag_2_CO_3_ during calcinations process. As seen from XRD patterns of the co-fired samples, only peaks of the metal and NaAgMoO_4_ phases were revealed, which means that both silver and aluminum are chemically compatible with the NaAgMoO_4_ ceramic at the sintering temperature. To study crystal structure details of the NaAgMoO_4_ ceramic, refinements were carried out using Fullprof software based on the fine XRD data. Both the situations of single and composite phases (spinel and silver) were considered. The observed and calculated XRD patterns are shown in Fig. 1 (b). The refined values of lattice parameters are a = b = c = 9.22039 Å, with a space group F D −3 M (227), according to the data (ICSD #159740) reported by Bouhemadou et al.^9^ The R_p_ = 12.1, R_wp_ = 14.8 and R_exp_ = 6.82 were obtained for single phase case as shown in Table I, while the R_p_ = 11.2, R_wp_ = 13.6 and R_exp_ = 6.79 were obtained for the composite phase and the ratio of silver phase is around 1.99%. In fact, due to the limited sensitivity of the XRD technique, the details of the remnant silver needs to be studied further. Schematic crystal structure of the NaAgMoO_4_ was presented in insert of Fig. 1 (b). Na and Ag cations arrange in the 8-coordinated site with a disordered distribution, while Mo cations occupy the 4-coordianted site.

Apparent densities of the NaAgMoO_4_ ceramics are shown in Fig. 2 (a). It is seen that as sintering temperature was increased from 350°C to 380°C, bulk density of the NaAgMoO_4_ ceramic increased from 4.45 to 4.77 g/cm^3^ and reached saturated at about 400°C with a relative density about 97% (the theoretical density calculated from XRD patterns was 4.928 g/cm^3^). SEM images of the NaAgMoO_4_ ceramic sintered at 400°C/2 h and BSE images of co-fired ceramics with 30 wt. % Ag are presented in the insert of Fig. 2. It is seen that dense and homogeneous microstructure can be observed from the as-fired surface while a few of pores can be observed from the fractured surface. The grain size scattered between 2 ~ 5 μm and both the as-fired and fractured surfaces correspond well with each other. The co-fired ceramics were found to be composed of both NaAgMoO_4_ and metal grains coupled with EDS analysis, which support the XRD results discussed above and further confirms that there were no intermediate phases and the desired chemical compatibility between NaAgMoO_4_ and silver powders was obtained.

Microwave dielectric permittivity and Qf value of the NaAgMoO_4_ ceramic as a function of sintering temperature are shown in Fig. 3 (a). As sintering temperature was increased from 350°C to 380°C, the dielectric permittivity increased from 7.0 to around 7.9 and kept stable. The Qf value first increased with sintering temperature, reached a maximum value at 400°C and then decreased slightly with further increase of sintering temperature. To further understand the temperature dependence of microwave dielectric properties of the NaAgMoO_4_ ceramic, the resonant frequency, the microwave dielectric permittivity and Qf value measured in the temperature range 20 ~ 125°C are demonstrated in Fig. S1 in the Supplementary Information. It can be seen that microwave dielectric permittivity of the NaAgMoO_4_ ceramic linearly increased with temperature slightly without any abnormity and temperature coefficient of resonant frequency is about −120 ppm/°C. The Qf value decreased slightly from 33,000 GHz at 20°C to 26,000 GHz at 125°C. In conclusion, the best microwave dielectric properties were obtained in ceramic sintered at 400°C with a permittivity ~7.9, a Qf value ~33,000 GHz and a temperature coefficient of resonant frequency ~−120 ppm/°C.

To further study the intrinsic microwave dielectric properties, infrared reflectivity spectra of the NaAgMoO_4_ ceramics were analyzed by using a classical harmonic oscillator model as follows: 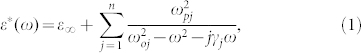
where ε*(ω) is complex dielectric function, ε_∞_ is the dielectric constant caused by the electronic polarization at high frequencies, γ_j_, ω_oj_, and ω_pj_ are the damping factor, the transverse frequency, and plasma frequency of the j-th Lorentz oscillator, respectively, and n is the number of transverse phonon modes. The complex reflectivity R(ω) can be written as: 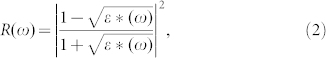
Fitted infrared reflectivity values, complex permittivities and phonon parameters are shown in Fig. 3 (b) and Table II. It is seen that the calculated dielectric permittivity and dielectric loss values are almost equal to the measured ones using TE_01δ_ method, which implies that majority of the dielectric contribution for this system at microwave region was attributed to the absorptions of structural phonon oscillation in infrared region and very little contribution was from defect phonon scattering. The optical dielectric constant calculated from the infrared spectra is about 3.07, which is almost 39% percent of the polarizability contribution at microwave region, and this implies that the contribution from the electronic polarizability can not be ignored in the low k (<10) microwave dielectric materials. The dielectric polarizability contribution of the strongest mode at 809.2 cm^−1^ is only 0.622, about 8% percentage, and this is due to its much higher frequency than the microwave region. The calculated dielectric loss is almost the same with the measured value and this means that there is no much space for the increase of Qf value by improving sintering process. The small microwave dielectric permittivity of NaAgMoO_4_ ceramic can also be explained by the Shannon's additive rule. At microwave region, the polarizability is the sum of both ionic and electronic components. Shannon^10^ suggested that molecular polarizabilities of complex substances could be estimated by summing the polarizabilities of constituent ions. Then the polarizabilities α could be obtained as follows: 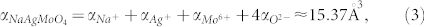
where the ionic polarization of Ag^+^ was set to be 2.25 Å^3^ as suggested in our previous work^11^. The ionic polarization of Na^+^, Mo^6+^ and O^2−^ were set to be 1.80 Å^3^, 3.28 Å^3^ and 2.01 Å^3^, respectively^10^,^12^. Considering the Clausius–Mosotti relation as follow: 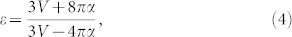
where the V is the cell volume, 783.88/8 Å^3^. The calculated dielectric permittivity is about 6.75, which is a little smaller than the measured value 7.9 and the extrapolated value 7.85. A 14.5 percent deviation of permittivity from the measured value is considered acceptable considering the simplicity of additive rule and the uncertainty of ionic polarization of Ag^+^.

## Summary

In conclusion, a novel NaAgMoO_4_ ceramic with high microwave dielectric performance, with a permittivity ~7.9, a Qf value ~33,000 GHz and a temperature coefficient of resonant frequency ~−120 ppm/°C, can be well densified at 400°C with grain size lying between 2 ~ 5 μm. From XRD and EDS analysis of the co-fired sample, it was found that the NaAgMoO_4_ ceramic is chemically compatible with both silver and aluminum at the sintering temperature and this makes it a candidate for the ultra-low temperature co-fired ceramics technology. Specifically, its densification temperature is almost half of that of the most popular low-fired Al_2_O_3_ material with glass addition and it might be promising in the dielectric substrate application.

## Methods

Reagent-grade Na_2_CO_3_, Ag_2_CO_3_, and MoO_3_ (>99%, Fuchen Chemical Reagents, Tianjin, China) were weighted according to the stoichiometric formulation NaAgMoO_4_. Powders were mixed and milled for 4 h by using a planetary mill. The powder mixture was then dried and calcined at 350°C for 4 h. The calcined powders were ball milled for 5 h to obtain fine powders. Then the powders were pressed into cylinders (10 mm in diameter and 4 ~ 5 mm in height) at 100 MPa. Samples were sintered at temperatures from 350°C to 420°C for 2 h. Room temperature X-ray diffraction (XRD) was performed by using a XRD with Cu Kα radiation (Rigaku D/MAX-2400 X-ray diffractometry, Tokyo, Japan). Diffraction pattern was obtained between 2θ of 10–80° at a step size of 0.02°. The results were analyzed by the Rietveld profile refinement method, using FULLPROF program. As-fired and fractured surfaces were observed by using a scanning electron microscopy (SEM, FEI, Quanta 250 F). Room temperature infrared reflectivity spectra were measured using a Bruker IFS 66 v FTIR spectrometer on Infrared beamline station (U4) at National Synchrotron Radiation Lab. (NSRL), China. The dielectric behaviors over 0.2 to 1.2 THz (6.7–40 cm^−1^) were measured by a terahertz time-domain (THz TDS) spectroscopy (ADVAVTEST TAS7500SP, Japan). A passive mode-lock fiber laser is used to pump and gate respectively two GaAs photoconductive antennas for the generation and detection of THz wave. Dielectric properties at microwave frequency were measured with the TE_01δ_ dielectric resonator method with a network analyzer (HP 8720 Network Analyzer, Hewlett-Packard) and a temperature chamber (Delta 9023, Delta Design, Poway, CA). The temperature coefficient of resonant frequency TCF (τ*_f_*) was calculated with the following formula: 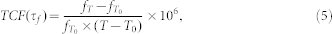
where *f*_T_ and *f*_T0_ are the TE_01δ_ resonant frequencies at temperature T and T_0_, respectively.

## Author Contributions

D.Z. and L.X.P. prepared the ceramic samples used in the measurement. D.Z. performed experiments and analyzed the data. Z.M.Q. and B.B.J. performed the far infrared reflectivity spectroscopy and THz dielectric spectroscopy, respectively. D.Z., L.X.P. and X.Y. discussed the results and contributed to the manuscript. All authors reviewed the manuscript.

## Supplementary Material

Supplementary Information SUPPLEMENTARY INFO

## Figures and Tables

**Figure 1:**
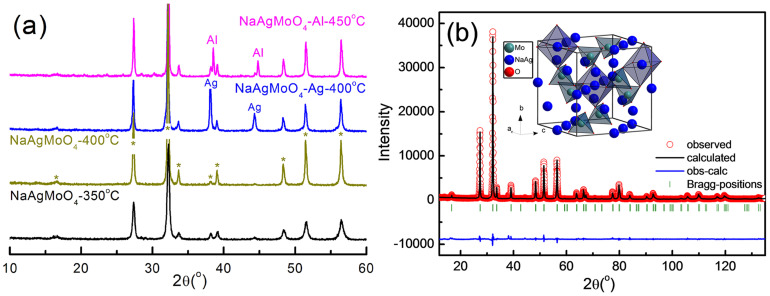
X-ray diffraction patterns of the NaAgMoO_4_ sample calcined at 350°C/4 h, 400°C/4 h, co-fired ceramic sample with 30 wt. % silver at 400°C/4 h and 30 wt. % aluminum at 450°C/4 h (a), and the experimental (circles) and calculated (line) X-ray powder diffraction profiles for NaAgMoO_4_ sample, which was though as a single phase (b) (R_p_ = 12.1, R_wp_ = 14.8 and R_exp_ = 6.82. The short vertical lines below the patterns mark the positions of Bragg reflections. The bottom continuous line is the difference between the observed and calculated intensity).

**Figure 2:**
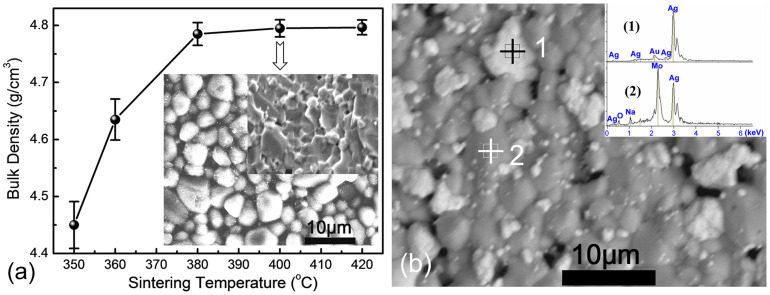
Bulk density of the NaAgMoO_4_ ceramic as a function of sintering temperature, and SEM image of NaAgMoO_4_ ceramic sintered at 400°C/2 h (a), BSE image of co-fired sample with 30 wt.% Ag at 400°C/4 h and EDS results (b).

**Figure 3:**
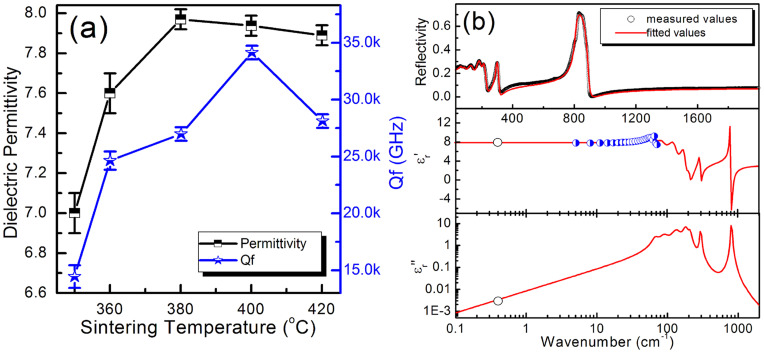
Microwave dielectric permittivity and Qf value of the NaAgMoO_4_ ceramic as a function of sintering temperature (a), the measured and calculated infrared reflectivity spectra (solid line for fitting values and circle for measured values) and complex dielectric spectra of NaAgMoO_4_ ceramic (b) (circles are experimental at microwave region and THz data, solid lines represent the fit of IR spectra).

**Table 1:** Refined atomic fractional coordinates from XRD data for NaAgMoO_4_ sample and the lattice parameters at room temperature are a = b = c = 9.22039 Å. The space group is F D −3 M (227)

Atom	Site	Occ.	x	y	z	Biso
Mo	8a	0.042	0.12500	0.12500	0.12500	0.534
Na	16d	0.042	0.50000	0.50000	0.50000	1.197
Ag	16d	0.042	0.50000	0.50000	0.50000	1.197
O	32e	0.167	0.23460	0.23460	0.23460	0.777

**Table 2:** Phonon parameters obtained from fitting of the infrared reflectivity spectra of NaAgMoO_4_ ceramic

Mode	*ω_oj_*	*ω_pj_*	*γ_j_*	Δ*ε_j_*
1	68.184	39.665	16.946	0.338
2	91.072	75.964	30.928	0.696
3	134.830	156.640	43.058	1.350
4	181.560	191.420	33.449	1.110
5	208.990	127.450	24.380	0.372
6	295.420	160.830	15.216	0.296
7	809.200	638.130	19.468	0.622
*ε*_∞_ = 3.070		*ε*_0_ = 7.854		
