# Supplementary material for: Novel ultra-low temperature co-fired microwave dielectric ceramic at 400 degrees and its chemical compatibility with base metal
Source: Sci Rep. 2014 Aug 7;4:5980. doi: 10.1038/srep05980 (PMC4124464; doi:10.1038/srep05980)
Supplement: Supplementary Information — SUPPLEMENTARY INFO [file srep05980-s1.pdf]

Title: Novel ultra-low temperature co-fired microwave dielectric ceramic at 400 degrees and its chemical compatibility with base metal

Authors: Di Zhou, Li-Xia Pang, Ze-Ming Qi, Biao-Bing Jin, and Xi Yao

It can be seen that the microwave dielectric permittivity of $NaAgMoO_4$ ceramic linearly increased with the temperature slightly without any abnormity. The Qf value decreased slightly from 33,000 GHz at 20 ◦C to 26,000 GHz at 125 ◦C. The temperature coefficient of resonant frequency is around -120 ppm/^{o}C. In conclusion, the best microwave dielectric properties were obtained in ceramic sintered at 400°C with a permittivity ~ 7.9, a Qf value ~ 33,000 GHz and a temperature coefficient of resonant frequency ~ -120 ppm/^{o}C.

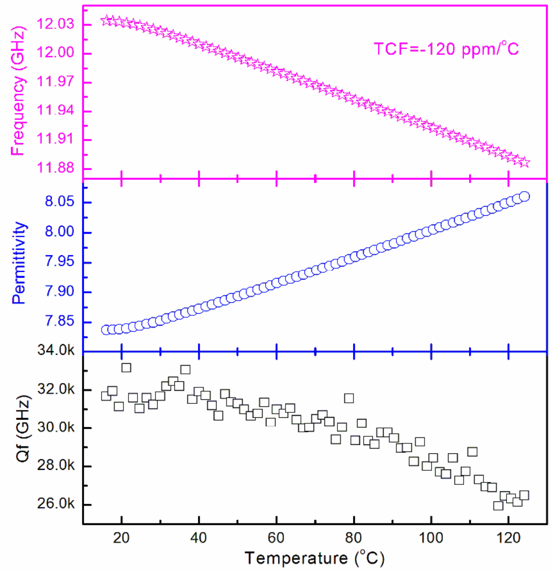

Fig. S1. Resonant frequency, dielectric permittivity and Qf value of $NaAgMoO_4$ ceramic sintered at 400 ^{o}C/2h in the temperature range of 20 ~ 125 ^{o}C
